# Light in the Rational Treatment of Autism? Effects of Metformin on Steroid Hormones in a Patient with Polycystic Ovarian Syndrome (PCOS)

**DOI:** 10.3390/life12111736

**Published:** 2022-10-28

**Authors:** Benedikt Gasser, Anca-Elena Calin, Genevieve Escher, Johann Kurz, Aglaia Emmenegger, Samuel Buerki, Arno Schmidt-Trucksäss, Markus Mohaupt

**Affiliations:** 1 Department of Sport, Exercise and Health, Division Sport and Exercise Medicine, University of Basel, Grosse Allee 6, CH-4052 Basel, Switzerland; 2 Lindenhofgruppe—Teaching Hospital of Internal Medicine, Lindenhofgruppe, CH-3006 Bern, Switzerland; 3 Department of Biomedical Research, University of Bern, CH-3008 Bern, Switzerland; 4 Interscience Research Collaboration, 8430 Leibnitz, Austria

**Keywords:** androgens, oxidative stress, mitochondrial function

## Abstract

**Background**: Metformin is an effective treatment option for type 2 diabetes mellitus, and it is, to this day, the most prescribed oral antiglycaemic drug. Besides its effects mainly on mitochondrial activity, an off-label use came up as a pharmaceutical for subjects with a diagnosis of polycystic ovarian syndrome (PCOS) along with altered steroid hormone homeostasis. Besides these effects, even an influence on mood and social behavior was described, leading to the aim of this case report to elucidate the effects before versus after treatment with metformin on steroid hormones and social behavior. **Methods**: A female patient with diagnosed PCOS was analyzed three times for steroid hormone levels. The first analysis was performed before treatment; the second, after a period of 71 days with metformin at 2 × 500 mg; and the third, after a total of 144 days with metformin at 2 × 500 mg. Spot urine probes were taken in the morning for a combined gas chromatography–mass spectrometry (GC-MS), and the steroid levels were adjusted for creatinine excretion. A questionnaire on social behavior (Autism Spectrum Questionnaire) was administered before treatment and after 71 days. **Results**: A decrease in all the steroid hormones measured was detected after 71 and 144 days of treatment with metformin, being more pronounced after 144 days of treatment and highly significant (*p* < 0.001). Furthermore, in the untreated state, the class of corticosterone metabolites showed increased values compared to the female reference values for TH-11-DH-corticosterone, TH-corticosterone, and 5a-TH-corticosterone. In the class of estrogen metabolites, increased values compared to the reference values were detected for 17b-estradiol; in the class of 11-deoxycortisol metabolites, an increase in TH-11-deoxycortisol was detected. For the class of cortisol metabolites, increased values compared to the reference values were detected for cortisone, TH-cortisone, a-cortolone, b-cortolone, 20b-dihydrocortisone, cortisol, TH-cortisol, 5a-TH-cortisol, a-cortol, 20b-dihydrocortisol, and 6b-OH-cortisol. No increases in androgen metabolites were detected. Interestingly, weight decreased from 93.4 kg to 91.3 kg after 71 days and fell to 82.7 kg after 144 days of treatment. The skeletal muscle mass was 30.1 kg at the first visit, decreasing to 29.9 kg and to 27.5 kg. No significant difference in the social behavior score from baseline to after 71 days of treatment was detected. **Discussion**: Metformin improved the steroid hormone profiles from levels above the upper reference values to the middle of the reference values after 71 days and to the lower ends of the reference values after 144 days of treatment. This implies not only that metformin has an effect on steroid hormone levels, but in addition that the efficacy of the pharmaceutical seems to depend on the time interval from intake. To summarize, in this patient, steroid hormones were affected but social behavior was not. If no effect of metformin on social behavior exists, this must be supported by further cases.

## 1. Introduction

Metformin is the first-line pharmacologic treatment for type 2 diabetes and the most commonly prescribed drug for this condition worldwide, used either alone or in combination with insulin or other glucose-lowering therapies [1]. Metformin is a biguanide, a drug class of herbal origin that has been widely used to treat diabetes since the 1950s [1]. The side effects include gastrointestinal problems and lactic acidosis; however, these are seldom severe, making this drug an excellent first-line treatment for diabetes [2,3,4,5]. Metformin reduces the glucose production in liver cells through an inhibition of the complex I in the mitochondria, while yielding further activation of AMPK (AMP-activated protein kinase), even further reducing the gluconeogenesis in liver cells [6]. Furthermore, AMPK activity has an antioxidant effect, resulting in reductions in both oxidative damage accumulation and chronic inflammation [7,8,9,10]. This leads to a reduction in oxidative stress, which is present in several illnesses ranging from genetic disorders and cancer to neurodevelopmental disorders with social withdrawal, even in the most severe form of autism [11,12,13]. Interestingly, ongoing clinical studies exist for the treatment of fragile-X-syndrome (FXS) (the most common single-gene cause of autism), and this might lead the way in targeted treatments for autism, especially as animal studies on FXS have demonstrated a hyperactive insulin receptor [14,15,16,17,18,19]. In consequence, elucidating the effect of metformin on steroid hormones seems fruitful for deepening our understanding of the biochemical mechanisms targeted by this pharmaceutical, allowing the development of rational recommendations on the usage of this pharmaceutical.

## 2. Materials and Methods

### 2.1. Case Participant

A 32-year-old female patient suffered from adiposity of WHO Grade I, with a body mass index (BMI) of 34.1 kg/m^2^, dyslipidemia, and polycystic ovarian syndrome (PCOS) diagnosed by a gynecologist. At the first appointment, we performed exhaustive cardiopulmonary exercise testing on a cycle ergometer, starting at 20 watts and ending up at 202 watts. The heart rate increased from 69/min to 179/min, the lactate concentration was 6.27 mmol/L at the end, the Borg Scale value was 19 at the end, and the ECG did not show any abnormalities. The measured physical activity measured with a wrist-worn accelerometer (GeneActive Activinsights Ltd., Kimbolton, UK) showed a pattern of average physical activity. The anthropometric measurements included a measurement of height and a four-segment bioelectrical impedance analysis using the InBody 720 (InBody Co., Ltd., Seoul, Korea). Furthermore, to capture social behavioral aspects, a questionnaire was administered, which is normally used in the autistic context, entailing 50 questions on social behavioral aspects [20]. Furthermore, this questionnaire was developed by researchers addressing steroid hormones directly for the understanding of social impairment [20]. The questionnaire was administered at the first appointment and the second, but not at the third appointment due to the patients’ wishes. After discussing the situation with the patient, we recommended increasing the physical activity from one to two times a week to three to five times per week (altering the intensity with sometimes more intense phases), recommended therapy with 2 × 500 mg of metformin, and took a spot urine probe in the morning for encompassing steroid hormone analysis with gas chromatography–mass spectrometry (GC-MS) as previously described [21]. A second appointment was made after 71 days of treatment, and a third appointment was made after 141 days, with the aim of losing weight and improving the monthly bleeding cycle.

### 2.2. Statistical Analysis

The sum of all the steroid hormone metabolites for each measurement was calculated. As Kolmogorov–Smirnov tests for each measurement indicated, at the level of *p* < 0.01, that the data were not normally distributed, Friedman tests were performed to detect differences in steroid hormone levels between the three measurement points. The calculations were performed with GraphPad Prism (GraphPad Software, Inc., La Jolla, CA, USA) and Microsoft Excel (Microsoft Inc., Redmond, WA, USA).

## 3. Results

The effects on anthropometry were impressive in terms of fat reduction while preserving muscle mass. For the weight at the first appointment, 93.4 kg was measured; after 71 days, 91.3 kg; after 144 days, 82.7 kg. The skeletal muscle mass was 30.1 kg at the first visit and decreased slightly to 29.9 kg at the second and to 27.5 kg at the third visit. The body fat share decreased from 42.1% to 41.6% after 71 days and to 40.0% after 144 days.

**Figure 1:**
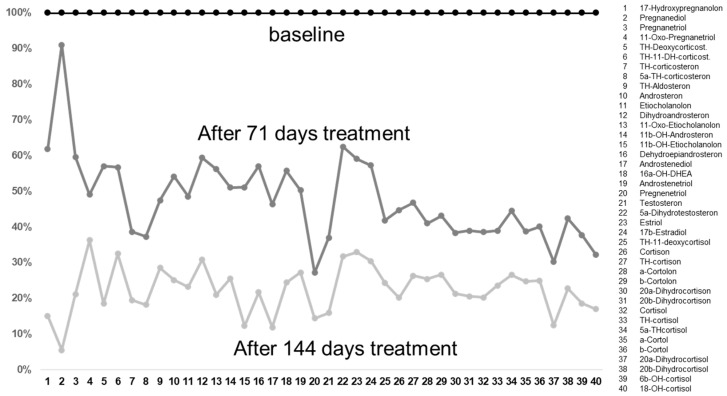
The decreases in the metabolites after 71 and 144 days of treatment with metformin compared to baseline values taken before treatment. It is interesting that, before the treatment, these were often already in the normal ranges, with some too-high exceptions (see Table 1). After treatment, these reached the lower ranges of the reference values. It is interesting that there was a dose dependence, in that the values decreased with an increasing duration of treatment.

The effects on steroid hormones are shown in Table 1 and Figure 1, where the decrease in androgens was the most prominent. As it was revealed by the Kolmogorov–Smirnov test for the first measurement (the K–S test statistic (D) was 0.287), for the second (the K–S test statistic (D) was 0.379), and for the third (the K–S test statistic (D) was 0.379) that the steroid hormone levels were not normally distributed (all three measurements, *p* < 0.001), the Friedman test was performed. Highly significant differences (*p* < 0.001) for the steroid hormone levels between the three measurement points were revealed by the Friedman test (the *X*^2^ statistic was 55.95 (2, N = 41)).

Interestingly, the levels of most of the cortisol and corticosterone metabolites were increased; increased values compared to the reference values were detected for TH-11-DH-corticosterone, TH-corticosterone, and 5a-TH-corticosterone. In the class of estrogen metabolites, increased values compared to the reference values were detected for 17b-estradiol; in the class of 11-deoxycortisol metabolites, an increase in TH-11-deoxycortisol was detected. For the class of cortisol metabolites, increased values compared to the reference values were detected for cortisone, TH-cortisone, a-cortolone, b-cortolone, 20b-dihydrocortisone, cortisol, TH-cortisol, 5a-TH-cortisol, a-cortol, 20b-dihydrocortisol, and 6b-OH-cortisol. However, not one single metabolite from the androgen class was increased.

As mentioned, the questionnaire on social behavior was administered twice at the first and the second visit, but not at the third visit, for personal reasons for the patient. The answer pattern is shown in Figure S1. Concerning social behavior measured with the Autism Questionnaire, no change was detected, with a total score of 15 points occurring twice; a subject without autism normally has a score of 16.4 points [2,20].

## 4. Outlook

We show here a case with a combined GC-MS before treatment and decreased levels of steroid hormones after 71 and even more pronounced after 144 days of therapy with metformin at 500 mg twice a day. Interestingly, in parallel with the decrease in steroid hormones, the body weight and, to a smaller extent, skeletal muscle mass decreased. The androgen levels, normally described as characteristically increased in subjects with PCOS, were not higher in this patient compared to the reference values [22]. As indicated from the cardiopulmonary exercise testing, the physical state of the patient was good [23], allowing the suggestion that a mild form of PCOS was present here, with androgen levels remaining within the reference range. The effect of metformin became stronger the longer the therapy continued. The effect was the lowest for pregnanediol (a progesterone metabolite) and the highest for pregnenetriol (an androgen metabolite). This seems to be in line with the favorable use of metformin for PCOS mainly targeting androgens [24,25,26,27]. Here, the effects on both steroid hormones and weight and body composition were pronounced. Of course, the findings are limited by the fact that only one case is presented. Nevertheless, a previously conducted power analysis according to the findings of Morin-Papunen et al., 2003, indicates that, at the single-metabolite level, significant effects are to be expected for even a sample size of seven patients [2,28]. In principle, metformin has a half-life of 6–8 h and, in consequence, a steady state is reached after around 30–40 h (after five half-lives—as a rule of thumb, the distribution volume is saturated after five half-lives; it does not imply that the pharmaceutical has the maximum effect) [29]. When focusing on reasons for the increased steroid hormones, oxidative stress might be causative, which might result from an increased 17,20-lyase-catalyzing activity of adrenal P450c17 through p38α [11,30,31,32,33]. It was, in consequence, indicated that these actions contribute to the beneficial effects of metformin [8,34]. Concerning mood and social behavior, it is important to mention that no effect was detected in this case, which stands in contrast to findings from a behavioral mouse model [34,35]. As, to date, neither the exact causes nor the biochemical basis of autism have been conclusively elucidated, the treatment options are not yet exhausted. However, we know from analyses of children with autism that there appears to be a change in the cholesterol pathway, yielding altered steroid hormones [30,31,32,33]. Focusing on therapeutic aspects, Bradstreet et al. had already, ten years ago, proposed spironolactone for autism in order to target steroid hormones [36], and metformin was considered in the treatment of the side-effects of antipsychotics in autism [37]. Interestingly, in a handful of patients being treated for FXS, an improvement in overeating but also in irritability, aggression, and social avoidance was detected [14,15,16,17,18,19]. As already mentioned, the mechanism might involve a hyperactive insulin receptor, which is intuitive, as postmortem studies have shown that the fragile mental retardation protein, the protein that is missing in FXS, is also deficient in the brain in patients with idiopathic autism without a fragile X mutation [14,15,16,17]. As insulin is closely related to steroid hormones, especially with glucocorticoids, a line of reasoning can be developed [14,15,16,17]. Furthermore, individual case studies have shown that macroorchidism did not develop in boys who started metformin clinically before puberty [38], and two adults with FXS improved their IQ when using metformin for over one year [39]. However, this case of a first patient analyzed with combined GC-MS remains vague concerning the effect of metformin on social behavior. In consequence, the usability of metformin for disorders such as autism remains vague. Further subjects need to be analyzed to come to a valid conclusion if a clinical trial is meaningful and metformin is a real treatment opportunity for disorders such as autism [2].

## Figures and Tables

**Table 1:** A combined GC-MS was performed at the first visit (baseline measurement) with urine and after 71 days of treatment at the second visit, and a third appointment was made after 141 days of treatment with metformin.

			First Visit	Second Visit	Third Visit	Reference
	Metabolite	Abbreviation	(µg/24 h)	(µg/24 h)	(µg/24 h)	(µg/24 h)
Progesterone metabolites	17-Hydroxypregnanolone	17-HP	189.0	231.0	74.5	21–254
	Pregnanediol	PD	312.0	2941.0	188.6	72–1411
	Pregnanetriol	PT	655.0	621.0	342.9	150–806
	11-Oxo-Pregnanetriol	PTONE	12.0	3.0	8.5	6–36
Corticosterone metabollites	TH-Deoxycorticost.	THDOC	19.0	17.0	8.2	2–21
	TH-11-DH-corticost.	THA	335.0	187.0	250.5	37–142
	TH-corticosterone	THB	298.0	93.0	94.4	54–206
	5a-TH-corticosteron	5a-THB	640.0	194.0	185.2	78–338
Aldosterone metabolites	TH-aldosterone	THALDO	25.0	9.0	13.6	6–30
Androgen metabolites	Androsterone	ANDRO	1556.0	982.0	850.4	184–1705
	Etiocholanolone	ETIO	1364.0	670.0	614.7	279–1825
	Dihydroandrosterone	DH-ANDRO	30.0	21.0	22.8	9–54
	11-Oxo-etiocholanolone	11-OXO-ETIO	430.0	344.0	205.5	109–616
	11b-OH-androsterone	11b-OH-ANDRO	1487.0	772.0	776.1	259–827
	11b-OH-etiocholanolone	11b-OH-ETIO	429.0	340.0	107.6	63–633
	Dehydroepiandrosterone	DHEA	22.0	18.0	11.1	14–374
	Androstenediol	ANDRO-DIOL	36.0	23.0	8.0	14–144
	16a-OH-DHEA	16a-OH-DHA	102.0	72.0	56.1	20–398
	Androstenetriol	5-AT	130.0	60.0	71.2	51–399
	Pregnenetriol	5-PT	138.0	24.0	27.3	8–253
	Testosterone	TESTOSTERONE	21.0	7.0	5.3	3–25
	5a-Dihydrotestosterone	5a-DIHYDROTEST	22.0	18.0	18.5	3–28
Estrogen metabolites	Estriol	ESTRIOL	11.0	7.0	8.8	1–22
	17b-Estradiol	17b-ESTRADIOL	8.0	5.0	5.7	1–7
11-Deoxycortisol metabolites	TH-11-deoxycortisol	THS	123.0	37.0	51.2	27–89
Cortisol metabolites	Cortisone	CORTISONE	248.0	110.0	90.3	74–234
	TH-cortisone	THE	5684.0	2180.0	2810.7	1162–3430
	a-cortolone	a-CORTOLONE	1874.0	495.0	805.6	517–1397
	b-cortolone	b-CORTOLONE	832.0	240.0	388.7	222–632
	20a-Dihydrocortison	20a-DHE	29.0	8.0	10.0	9–30
	20b-Dihydrocortison	20b-DHE	90.0	27.0	30.3	27–86
	Cortisol	CORTISOL	198.0	59.0	65.1	43–160
	TH-cortisol	THF	3330.0	836.0	1287.1	672–1968
	5a-TH-cortisol	5a-THF	2227.0	718.0	1061.8	266–1369
	a-cortol	a-CORTOL	433.0	99.0	174.5	125–372
	b-cortol	b-CORTOL	481.0	121.0	200.3	164–501
	20a-Dihydrocortisol	20a-DHF	67.0	17.0	11.9	22–93
	20b-Dihydrocortisol	20b-DHF	121.0	41.0	47.7	23–111
	6b-OH-cortisol	6b-OH-F	264.0	81.0	78.6	41–191
	18-OH-cortisol	18-OH-F	552.0	124.0	137.9	66–380
	Total		24,824.0	12,852.0	11,207.2	

## Data Availability

Data is available on qualified request to the corresponding author.

## Supplementary Materials

The following supporting information can be downloaded at: https://www.mdpi.com/article/10.3390/life12111736/s1. Figure S1: No effect on the total score was detected, which was, at baseline measurement, 15 points and, after 71 days of treatment with metformin twice a day at 500 mg, once again, 15 points. The average score for a patient without autism is a score of 16.4, indicating no hints for autistic traits in this patient (2), (14). In detail, the answer class definitely disagree was increased, whereas slightly disagree decreased from baseline measurements (light gray) to the measurement after treatment (dark gray).
