# Supplementary material for: Light in the Rational Treatment of Autism? Effects of Metformin on Steroid Hormones in a Patient with Polycystic Ovarian Syndrome (PCOS)
Source: Life (Basel). 2022 Oct 28;12(11):1736. doi: 10.3390/life12111736 (PMC9696325; doi:10.3390/life12111736)
Supplement: Supplementary file 1 [file life-12-01736-s001.zip › life-1930469-supplementary.pdf]

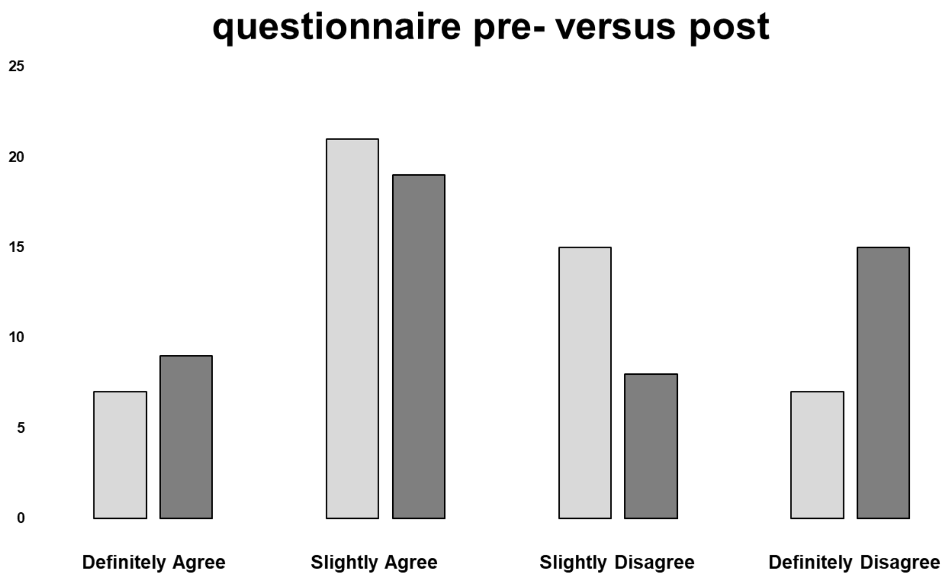

Figure S1. No effect on the total score was detected, which was, at baseline measurement, 15 points and, after 71 days of treatment with metformin twice a day at 500 mg, once again, 15 points. The average score for a patient without autism is a score of 16.4, indicating no hints for autistic traits in this patient (2), (14). In detail, the answer class definitely disagree was increased, whereas slightly disagree decreased from baseline measurements (light gray) to the measurement after treatment (dark gray).
